# Radioguided surgery with *β*^−^ radiation in pancreatic Neuroendocrine Tumors: a feasibility study

**DOI:** 10.1038/s41598-020-61075-2

**Published:** 2020-03-04

**Authors:** Francesco Collamati, Daria Maccora, Sergio Alfieri, Valerio Bocci, Antonella Cartoni, Angela Collarino, Micol De Simoni, Marta Fischetti, Ilaria Fratoddi, Alessandro Giordano, Carlo Mancini-Terracciano, Riccardo Mirabelli, Silvio Morganti, Giuseppe Quero, Dante Rotili, Teresa Scotognella, Elena Solfaroli Camillocci, Giacomo Traini, Iole Venditti, Riccardo Faccini

**Affiliations:** 1 INFN Sec. of Rome, P.le A. Moro 2, 00185 Rome, Italy; 2 Physics Dep. of “Sapienza” University, P.le A. Moro 2, 00185 Rome, Italy; 3 SBAI Dep. of “Sapienza” University, P.le A. Moro 2, 00185 Rome, Italy; 4 Nuclear Medicine Unit, Fondazione Policlinico Gemelli IRCCS, L.go A. Gemelli 8, 00168 Rome, Italy; 5 Centro Studi e Ricerche E. Fermi, Rome, Italy; 6 Università Cattolica del Sacro Cuore, L.go F. Vito 1, 00168 Rome, Italy; 7 Specialty School of Medical Physics of “Sapienza” University, P.le A. Moro 2, 00185 Rome, Italy; 8 Chemistry Dep of “Sapienza” University, P.le A. Moro 2, 00185 Rome, Italy; 9 Sciences Dep. of “Roma Tre” University, Viale G. Marconi 446, 00146 Rome, Italy; 10 Department of Chemistry and Technologies of Drugs of “Sapienza” University, P.le A. Moro 2, 00185 Rome, Italy; 11 Digestive Surgery Unit CRMPG, A. Gemelli Hospital IRCCS of Rome, Università Cattolica del Sacro Cuore, L.go F. Vito 1, 00168 Rome, Italy

**Keywords:** Preclinical research, Translational research, Oncology, Applied physics

## Abstract

The possibility to use *β*^−^ decaying isotopes for radioguided surgery (RGS) has been recently proposed, and first promising tests on *ex-vivo* samples of Meningioma and intestinal Neuroendocrine Tumor (NET) have been published. This paper reports a study of the uptake of ^68^Ga-DOTATOC in pancreatic NETs (pNETs) in order to assess the feasibility of a new RGS approach using ^90^Y-DOTATOC. Tumor and healthy pancreas uptakes were estimated from ^68^Ga-DOTATOC PET/CT scans of 30 patients with pNETs. From the obtained SUVs (Standardised Uptake Value) and TNRs (Tumor Non tumor Ratio), an analysis algorithm relying on a Monte Carlo simulation of the detector has been applied to evaluate the performances of the proposed technique. Almost all considered patients resulted to be compatible with the application of *β*^−^-RGS assuming to administer 1.5 MBq/kg of activity of ^90^Y-DOTATOC 24 h before surgery, and a sampling time of few seconds. In just 2 cases the technique would have required a mildly increased amount of activity or of sampling time. Despite a high physiological uptake of ^68^Ga-DOTATOC in the healthy pancreas, the proposed RGS technique promises to be effective. This approach allows RGS to find application also in pancreatic diseases, where traditional techniques are not viable.

## Introduction

Radio-Guided Surgery (RGS) is a procedure aimed at helping the surgeon to completely and precisely resect a tumour. It requires to administer a radio-labeled pharmaceutical, with a specificity to the tumor, to the patient before the surgery. Such a radio-pharmaceutical will be detected during the surgery with an handheld detector, hereinafter named *probe*, designed to reveal emitted radiation and, thus, allowing to identify the tumoral area, and its borders in particular.

Established methods use nowadays a combination of a *γ*-emitting radio-pharmaceutical with a *γ*-radiation-detecting probe^1,2^. The most common isotope is ^99*m*^Tc, that emits 144 keV photons with a *τ* = 8.7 h.

However, the use of *γ* rays constitutes itself a limitation to the applicability and efficacy of the technique. In fact, about 1/3 of such photons traverses more than 8 cm in human tissue, implying that the uptake of the compound in healthy tissues nearby lesions of interest constitutes a non-negligible background. As a result, *γ*-RGS is today particularly used in those contexts in which the target is located in low uptake regions (e.g. sentinel lymph node).

A recent prospective study^3^ has evaluated the possibility of performing *γ*-RGS in abdominal Neuroendocrine Tumors by using ^68^Ga-DOTATATE as radio-pharmaceutical, i.e. a *β*^+^ emitter. Despite overall positive conclusions, the authors highlight that out of the 25% (33/133) of false positive cases, the majority came from pancreas, liver and gastro-intestinal tract, i.e. organs characterised by an elevated level of physiological uptake of radio-pharmaceutical, or close to organs with a high normal uptake^3^.

In the view of overcoming these limitations, the use of pure *β*^−^-emitting rather than *γ*-emitting radio-pharmaceuticals can be considered. Indeed, electrons emitted in *β*^−^ decays have a mean free path of few millimetres in human tissues. Moreover, as the *bremsstrahlung* emission has a probability of 0.1% at these energies energies, the *γ* contamination can be considered negligible^4^.

All in all, the use of a radiation with such a small penetration would allow a higher spatial sensitivity and, hence, a clearer delineation of margins of the pathologic tissue with respect to *γ* emission^5^. In fact, when dealing with electrons, due to pure geometrical reasons, it is just the detector transverse size that determines its spatial resolution, i.e. the capacity to discriminate two adjacent sources, or to identify the tumor margin. In particular, the FWHM (Full Width at Half Maximum) of the scan profile is expected to be very close to the size of the source along the explored projection. Furthermore, the dose to the medical personnel becomes negligible too.

As a matter of fact, the idea of using *β*^−^ emitting isotopes was at the base of the very first inception of the Radio Guided Surgery technique itself^6^, that however eventually did not succeed into clinical practice due to factual limitations at the time in both the radio-pharmaceutical handling and the detector technology. In this context, we recently suggested the possibility of a new approach to *β*^−^-RGS^7–9^, starting with the development of a dedicated detector. Such detector features high sensitivity to *β* particles and substantial transparency to *γ* radiation, that constitutes a background in this context, together with the expected exquisite spatial resolution^10^.

This proposed novel technique of RGS proved not only to be theoretically possible^11,12^, but also its actual feasibility was demonstrated with ex-vivo tests on meningioma^13,14^, as well as gastro-entero-pancreatic Neuroendocrine Tumors (GEP-NET)^15,16^ samples, marked with ^90^Y-DOTATOC (pure *β*^−^ decay with an endpoint of 2.2 MeV and half-life *τ*_1∕2_ = 64 h).

In particular, the remarkable efficacy showed by *β*-RGS in GEP-NET ex-vivo tests, whose samples included an abundant tract of healthy bowel among which tumors had to be discriminated, enforces the expected adeptness of such a technique to locate lesions in a high background situation.

An excellent application case in this view can be represented by pNETs. These are in fact mildly common lesions, having a prevalence of 10%^17^, in which surgical excision is the gold standard treatment, and where the possibility to minimise the asportation of healthy pancreas could be of remarkable importance for the patient’s outcome. More specifically, pNETs enucleation implies a significantly minor invasiveness as compared to formal pancreatectomy, leading to a relevant tissue sparing and less incidence of long-term functional consequences (i.e. post-operative diabetes, exocrine insufficiency)^18^. Moreover, the pancreas thickness is few centimeters, compatible with the use of beta radiation, that could be otherwise obscured if emitted from tumors at greater depths. Lastly, as already discussed, this kind of tumor expresses receptors for somatostatine, thus allowing the use of ^90^Y-DOTATOC, as in previous aforementioned studies.

Therefore, the aim of this study is to evaluate the possibility to apply *β*-RGS to pancreatic NETs, using ^90^Y-DOTATOC as radio-pharmaceutical.

This technique would in fact improve the intra-operative localization and detection of pNETs, particularly difficult especially in case of small tumors^19^. For instance, this precise tumor localization would be of outmost importance to drive the resection, leading to a potential reduction of the current incidence of margin positivity after enucleation (R1 margins), reported in almost the 37% of pNETs located in the pancreatic head^20^.

To such a purpose, a retrospective study on PET images of patients affected by this pathology after administration of ^68^Ga-DOTATOC has been performed at Fondazione Policlinico Gemelli, Rome. Following the procedure reported in ref. ^11,21^, we used the measured uptake in tumor and nearby healthy tissues to study the sensitivity of a *β*^−^ probe to the signals of interest, thus evaluating the feasibility of the application of RGS in this case.

## Methods

### Patients population

Thirty patients who had a ^68^Ga-DOTATOC PET/CT between March 2015 and February 2017 and showing a pancreatic uptake likely to be a pNET were retrospectively identified. PET/CT scans were performed at the institute of Nuclear Medicine of Fondazione Policlinico Universitario A. Gemelli IRCCS in Rome. The Review Board of the clinical institute involved, namely: *Comitato Etico Fondazione Policlinico Universitario “Agostino Gemelli” - Università Cattolica del Sacro Cuore - Rome*, approved this study and all patients signed a written informed consent form.

### Imaging protocols and interpretation

#### ^68^Ga-DOTATOC PET/CT

The administered activity of ^68^Ga-DOTATOC was 1.5–3 MBq/kg (100–200 MBq), and imaging was acquired between 60 ±  10 min post intravenous injection. PET images were acquired for 2–2.5 min per bed position. The whole-body (from head to middle of the upper leg) PET/CT was performed on a hybrid scanner (Gemini GXL; Philips Medical Systems, Cleveland, Ohio and Biograph mCT, Siemens, Germany). A low-dose CT scan was first performed and then PET images of the same field of view were obtained. PET images were reconstructed using an iterative protocol and displayed on a dedicated workstation of transaxial, coronal and sagittal planes for anatomical reference and attenuation correction purposes^22,23^.

A line-of-response row-action maximum-likelihood algorithm (3 iterations and 33 subsets, voxel size of 4 × 4 × 4 mm, no additional gaussian smoothing) or a 3-dimensional (3D) ordered-subsets expectation-maximization algorithm with resolution modelling (2 iterations and 21 subsets, voxel size of 3.2 × 3.2 × 5 mm, additional gaussian smoothing of 2 mm in full width at half maximum) were used for the Gemini or Biograph, respectively.

#### Image interpretation

Two experienced nuclear medicine physicians interpreted visually PET/CT scans, analyzed and reviewed the datasets.

### Estimate of the DOTATOC uptake

In order to estimate the possibility to apply this technique, the SUV (Standardized Uptake Value) and TNR (Tumor Non tumor Ratio) of DOTATOC in pNETs are needed. This is possible exploiting PET exams performed with ^68^Ga-DOTATOC.

The SUV is a semi-quantitative measurement of the amount of radio-pharmaceutical uptake in a given region of interest, normalized to the administered activity and to body mass of the single patient. It is defined as:

1 $${\rm{S}}UV=\frac{\mu W}{{A}_{adm}{e}^{-0.693\Delta {t}_{PET}/{T}_{Ga}^{1/2}}},$$

in which *μ* is the mean value of PET activity of the considered area, *A*_*adm*_ is the amount of activity administered, *W* is the patient mass, Δ*t*_*PET*_ is the time between the radio-pharmaceutical administration and the PET scan, and ${T}_{Ga}^{1/2}$ is finally the ^68^Ga half-life. The Tumor Non tumor Ratio (TNR) is the ratio between the SUVs of the tumor and of the background healthy tissue.

**Figure 1:**
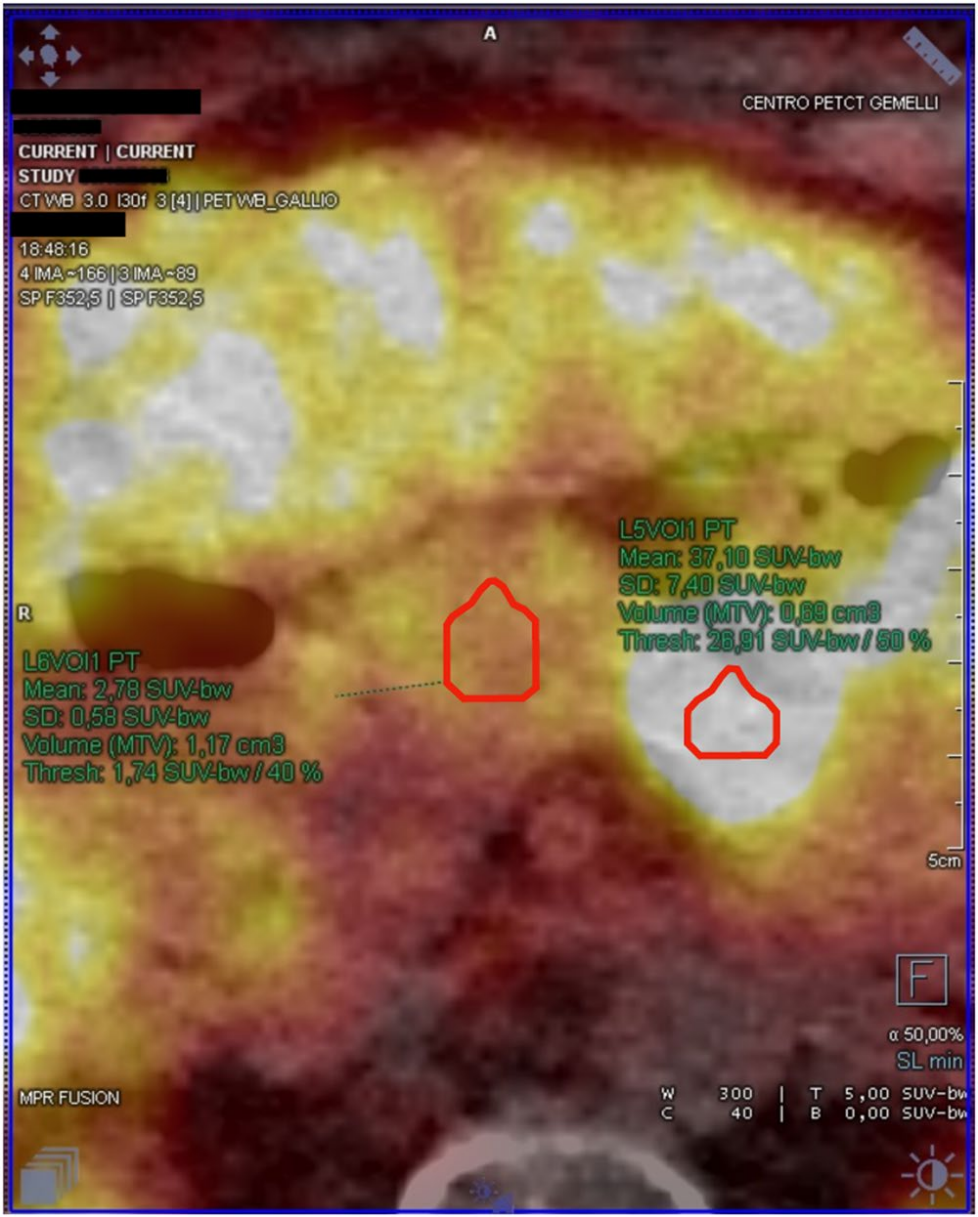
Example of ROI identification in the tumor (right ROI) and in the nearby healthy pancreas (left ROI).

Being the application case of this study the intraoperative discrimination of tumor margins and/or remnants after the bulk excision, we decided to choose as “tumor” the marginal proximity of the lesion, and as reference “healthy tissue” the adjacent area, as depicted in Fig. 1. This is in fact what the surgeon should be able to discriminate from the lesion in a hypothetical application case.

In order to obtain these uptake values, we examined ^68^Ga-DOTATOC PET scans of the patients in the cohort. The “3d-isocontour” tool was used to define ROIs (Regions Of Interest) throughout different slices in which to compute the mean uptake, thus forming VOIs (Volumes Of Interest). The threshold value for this ROI selecting tool was set to 50% of the maximum value, and the VOIs were chosen to have approximately a volume of 100 mm^3^ for homogeneity of comparison.

For each VOI, the mean SUV ($\bar{SUV}$), the SUV rms (Δ_*SUV*_) and the VOI volume (*V*) were measured. The error on the mean SUV value was then calculated as:

2 $${\sigma }_{\bar{SUV}}=\frac{{\Delta }_{SUV}}{\sqrt{N}},$$

where *N* is the number of voxels included in the VOI.

### The *β* probe

A fundamental element in the development of the *β*^−^-RGS technique is the detector to be used to reveal electrons. As detailed in previous studies^4,11,12,24^, we developed a *β* probe, that exploits the low penetration power of *β* particles by diminishing the thickness of the lateral shielding, resulting in a handy and light tool, when compared to commercial *γ* probes. The active element of the detector is made of *p-terphenyl*, an organic scintillator characterised by high light yield and low density^24^, that makes it an ideal choice to reach good sensitivity to *β* particles, while remaining substantially transparent to photons.

In particular, the probe prototype that has been tested on *β*^−^ radiation in a clinical trial with meningioma ex-vivo samples, and which is used in this study, is based on a sensitive cylinder of *p-terphenyl* with a radius of 2.55 mm and a 3 mm depth. To maximise the accuracy on the direction of the incoming radiation, this sensitive region is surrounded by a ring of 3 mm of polyvinylchloride. The scintillation light is transported then read by a Silicon Photo Multiplier sensor (SiPm, SenSL series C, mod. 10035), controlled and readout using ArduSiPM^25^.

It has to be mentioned that in the time elapsed since the meningioma campaign, several advances have been made regarding the detector, which has gained in sensitivity. However, for the sake of comparing these studies with the previously published ones, in this paper we use as reference the performances of the previous model of detector, as a conservative approach.

### Calculation of expected rates

Once obtained the uptake values from the images, it is necessary to quantify the performances of the probe in the real application scenario. The procedure is detailed in^26^, and essentially relies on a Monte Carlo simulation where the probe is exposed first to a small tumoral remnant encapsulated in a healthy tissue cylinder and then to an area of healthy tissue as well. Dimensions have been chosen to be 6 mm diameter - 7 mm height for the tumor remnant, thus mimicking a typical volume of a residual to be looked for during RGS, and 2 cm diameter - 1 cm height for healthy tissue. In fact, since ^90^Y electrons penetrate only few mm in human tissue, the “field of view” of the probe is limited to particles that originated from few mm in front of the active element, and extends transversely according to the horizontal dimension of the scintillator itself (in this case 2.55 mm radius). Therefore, further enlarging this “healthy tissue” volume in the Monte Carlo would not give additional signal to the probe, since no more particles able to reach the detector are created when generating at higher distances from it, but would only have impact on the computational time.

All in all, the mentioned simulation algorithm allows to obtain, from SUV and TNR of the considered tumor, the Rates in *cps* we expect to count on the lesion (*R*_*T*_) and on the nearby healthy tissue (*R*_*H*_) in a real application case. Evidently, the amount of injected activity is needed to perform this prediction, as well as the time interval between the injection and the performing procedure. These are indeed necessary parameters to predict the specific activity in the considered areas at the time when RGS is performed. In this study we assumed to inject 1.5 MBq/kg of ^90^Y-DOTATOC, which has been evaluated^14^ as the reasonable minimal value of activity to have good discrimination in the case of meningiomas, therefore using a reference total activity (for a 70 kg patient) of 105 MBq.

Moreover, the long enough physical decay time of ^90^Y (*τ* = 92 h) does not put particular operational constraints on the time interval between the injection and the procedure. We have demonstrated^12^ that the DOTATOC TNR in NET reaches a maximum at about 24 h post injection, due to the simultaneous action of receptor mediated accumulation and healthy organs’ wash out. It is thus possible to conceive an application protocol in which the patient is injected the day before surgery, thus maximising the efficacy of the technique, hence allowing in turn to diminish the activity to be injected.

From the so estimated *R*_*T*_ and *R*_*H*_, it is possible to compute the minimum sampling time (Δ*T*_*min*_) the detector needs to be on the area to be able to distinguish with sufficient accuracy tumor from healthy tissue.

To this aim, we applied in this study the same approach described in ref. ^7,21^, that is based on the computation of the rate of false positive (FP) and false negative (FN) signals. For a given value of the probe sampling time (*t*_*probe*_), the number of signal counts coming from the tumour and the background is distributed according to a Poisson distribution with mean *μ*_*T*_ = *R*_*T*_ × *t*_*probe*_ and *μ*_*H*_ = *R*_*H*_ × *t*_*probe*_ respectively. Given the minimum number of signal counts (*μ*_*th*_) needed to identify as positive a spot, FP is evaluated as the fraction of times the background would give a positive signal:

3 $$FP=1-{\sum }_{N=0}^{{\mu }_{th}-1}{P}_{{\mu }_{H}}(N),$$

where *P*_*μ*_(*N*) is the Poisson probability of having *N* if the mean is *μ*. In the same way, FN is the fraction of times a tumour residual would not yield an above threshold signal:

4 $$FN={\sum }_{N=0}^{{\mu }_{th}-1}{P}_{{\mu }_{T}}(N).$$

Intuitively, for a fixed *t*_*probe*_, rising the *μ*_*th*_ value would increase the number of healthy zones that are defined as “not tumor”, thus diminishing the FN rate. On the other hand, however, it would also rise the number of possible tumors that are flagged as “non tumor”, thus increasing the FP rate. The same reasoning stands for the case in which *μ*_*th*_ is fixed and *t*_*probe*_ is varied. To identify the minimum probing time, FN and FP are evaluated in a grid of *t*_*probe*_ and *μ*_*th*_ and the smallest value of *t*_*probe*_ for which *FN* < 5% and *FP* ≈ 1% is determined.

### ROC analysis

The described procedure allows to estimate the optimal probing time needed on each lesion. However, in the real application case, i.e. during the RGS procedure, clinical experience suggests that the time the surgeon will spend on each analysed area will be almost constant, and certainly can not depend on the unknown real activity of the lesion. First ex-vivo tests^15,16^ suggest that, independently from the sample uptake, the surgeon typically tends to stay on each spot at least ≈3s before being confident enough to determine if it is healthy or not.

Therefore, a sensitivity analysis has been performed to evaluate the discriminating power of the proposed technique, having assumed a fixed probing time of *t*_*probe*_ = 3s, and varying the threshold value (*th*) in terms of total number of counts obtained in this time interval. For each given threshold value, “sensitivity” and “specificity” were evaluated and plotted in a ROC curve, the area of which can be used to evaluate the discriminating power of the technique.

### Ethical approval

All procedures performed in studies involving human participants were in accordance with the ethical standards of the institutional and/or national research committee and with the 1964 Helsinki declaration and its later amendments or comparable ethical standards.

## Results

### Uptake and TNRs

**Figure 2:**
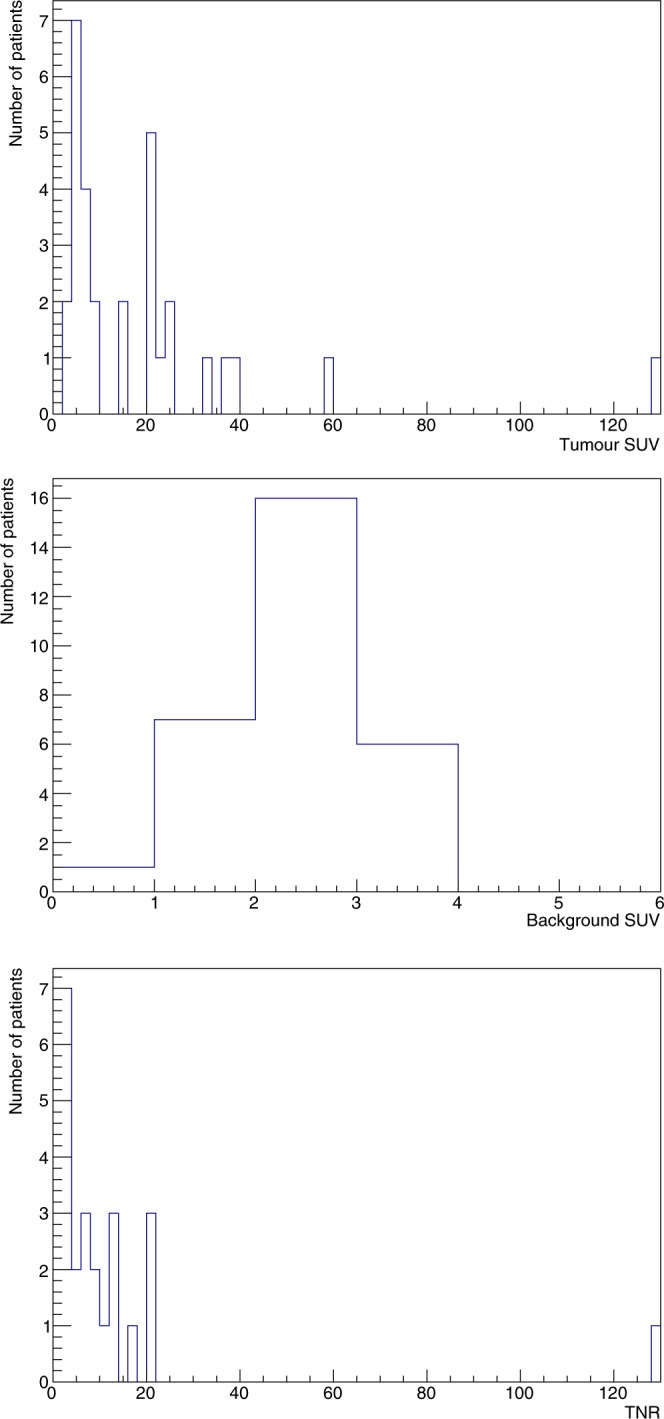
Distribution of tumor SUVs (top), healthy pancreas SUVs (middle) and TNRs (bottom) for all the 30 patients.

**Figure 3:**
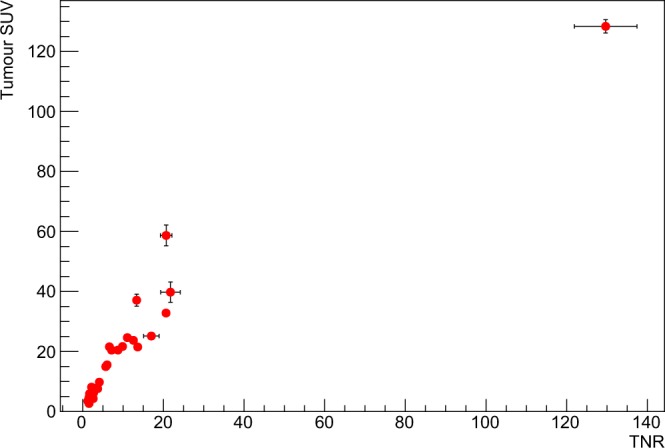
Scatter plot showing tumor SUV as a function of TNR for all the 30 patients. Errors shown in the bars are obtained as described in the text.

The described analysis procedure has been applied to all the 30 patients to obtain their respective SUVs and TNRs. Median SUV was 12.4 (InterQuartile Range 5.5–23.2) for tumors and 2.4 (IQR 1.9–2.9) for healthy pancreas, with a median TNR of 4.9 (IQR 2.2–12.2). Figure 2 shows SUVs (for both healthy pancreas and tumors) and TNRs histograms, while in Fig. 3 the scatter plot of tumor SUV versus TNR is shown.

### Sensitivity analysis

**Figure 4:**
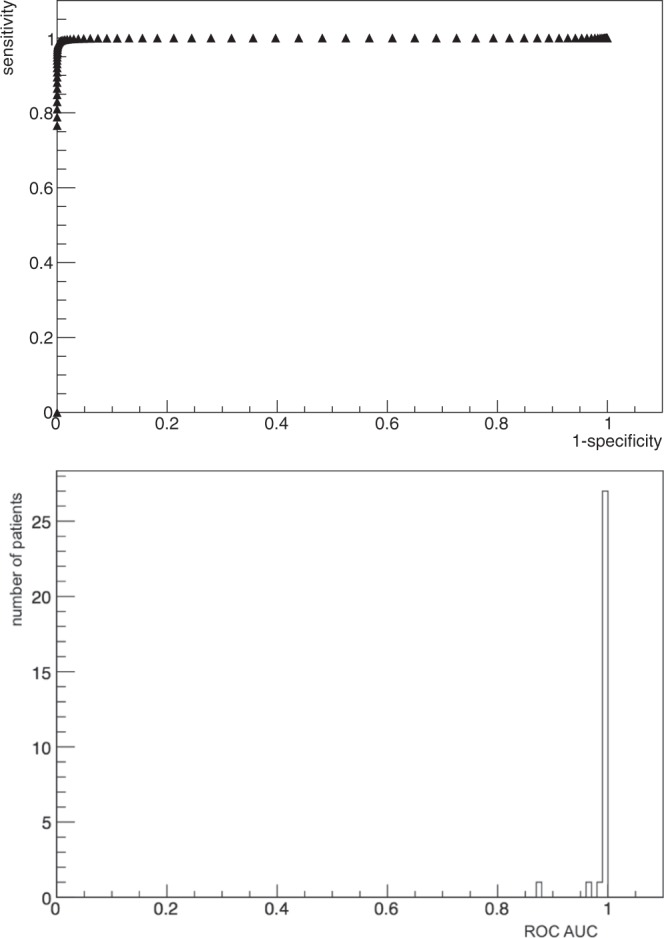
ROC curve for patient n. 1 (top), and distribution of Area Under Curve of ROCs of all the considered patients (bottom). The AUC for the shown case is 0.9999575. The probing time has been fixed to *t*_*probe*_ = 3 s.

  Figure 4 (top) shows the ROC curve for patient n. 1, obtained as described before in the text. Furthermore, Fig. 4 (bottom) shows the distribution of the Area Under Curve for all the considered 30 patients.

### Expected performances of RGS with *β* decays

**Figure 5:**
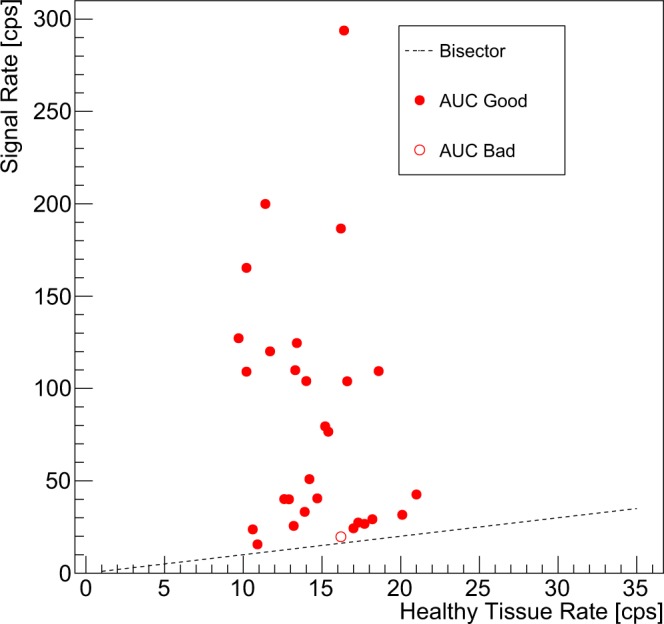
Counting rates expected on lesions (Signal) versus the ones expected on healthy tissue. The dashed line is the bisector, representing the case in which the probe gives the exact same count over signal than over healthy tissue, having thus no sensitivity at all. Points are classified (“Good” or “Bad”) according to the ROC sensitivity test described in the text, having fixed a probing time of 3 s and requiring *AUC* > 0.95. The rates correspond to the application case described in the text (injection of 105 MBq of ^90^Y-DOTATOC 24 h before the surgery).

Results for SUVs and TNRs shown in Figs. 2 and 3 have been used, following the procedure described in the previous paragraph, to obtain the counting rates we expect in a real application scenario. The results are shown in Fig. 5, where points are classified according to the ROC sensitivity test (“Good” if *AUC* > 0.95, “Bad” otherwise). In the plot, the bisector represents the “worst case”, in which tumor and healthy tissue give the same signal. Median probing time (*t*_*probe*_) was 0.4 s (IQR 0.1–1.7 s).

**Figure 6:**
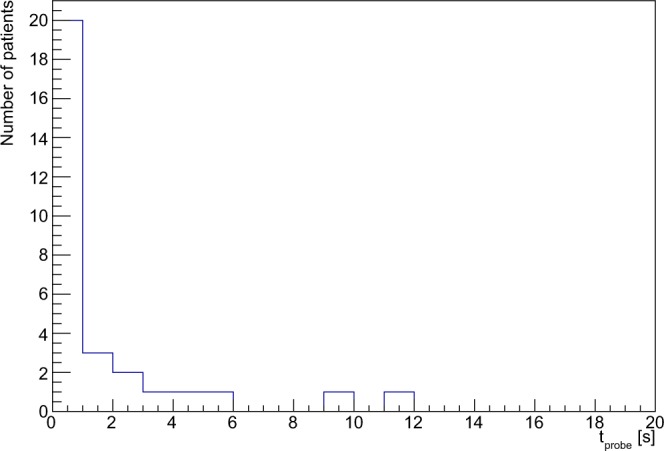
Distribution of time (*t*_*probe*_) needed to discriminate with sufficient accuracy the tumor. The statistical criterion used and the considered real case scenario are described in the text.

**Figure 7:**
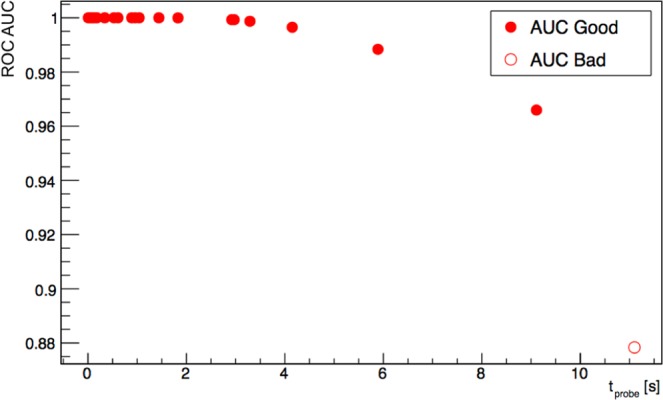
Area Under Curve of ROCs as a function of the probing needed time *t*_*probe*_ calculated for each of the 30 patients. Points are classified (“Good” or “Bad”) according to the ROC sensitivity test described in the text, having fixed a probing time of 3s and requiring *AUC* > 0.95.

  Figure 6 shows the detecting times needed to discriminate the tumor from the surrounding healthy tissue, obtained as described earlier in the text, while in Fig. 7 the correlation between the ROC-AUC and this detecting time is reported.

## Discussion

The goal of this study was to evaluate the possibility to apply an innovative *β*^−^-RGS technique to pancreatic Neuroendocrine Tumors with ^90^Y-DOTATOC, using a detector that has been recently developed and tested on Meningioma and bowel NET ex-vivo samples^13–16^.

This technique, with respect to enstablished *γ*-RGS, would allow a clearer delineation of margins of the pathologic tissue together with a substantial reduction of the dose given to the medical staff.

As expected, tumor SUVs and TNRs are found to vary significantly among patients, ranging from less than 2 in the lowest cases up to more than 100 for the highest one.

On the other hand, the SUV of healthy pancreas has been found to be more consistent among patients, showing smaller variations. It has however to be stressed that the average value of background SUV (median 2.4) has been found to be significantly higher than in previous studies dedicated to assessing the feasibility of the proposed *β*-RGS technique in other application cases (of the order of 0.2 for both brain tumors^11,12^ and prostate cancer^21^).

This was indeed an expected finding, since healthy pancreas is known for its elevated physiological uptake, that has hindered so far any application of traditional Radio Guided Surgery, i.e. exploiting *γ* decays, notwithstanding the availability of possible radio-pharmaceuticals like ^177^Lu-DOTATATE.

Nevertheless, the very good TNR showed by the vast majority of cases, together with the high sensitivity of the foreseen detector, that exploits the locality of beta particle emission, allows to perform the proposed technique with very high efficacy, as highlighted by both the ROC sensitivity test and the time needed to exploit the procedure.

In fact, Fig. 6 suggests that a probing time of few seconds is enough to identify all lesions. In our experience with ex-vivo tests performed so far, we found out that, independently from the actual probe signal, the operator tends to remain on each spot at least  ~ 3–5s, before being confident enough in his/her measurement. It has thus to be noted that only in 5 out of 30 cases the sampling time would slightly exceed this 3 s “reaction time”, while more than 93% of cases (28/30) would have been detected within 5 s.

Indeed, only 2 cases presented such a low TNR that would imply a needed probing time of about 10 s, which is probably an amount of time not compatible with surgical practice. These cases in fact correspond to points near the bisector in Fig. 5, representing occurencies in which tumor and healthy tissue uptake were almost similar. In this context, it has however to be noted that the application protocol of *β*-RGS is based on a preliminary PET study with ^68^Ga-DOTATOC, to be performed few weeks before surgery. This exams would be exactly dedicated to assess whether the considered lesion shows a TNR sufficient to make the patient an eligible candidate for such a technique, or whether an excessive amount of time or injected activity would be needed, thus making this technique not a viable approach.

Regarding radio protection issues, the use of *β*^−^ radiation implies, with respect to *γ*-RGS, a much reduced exposure for the medical personnel, that has in fact found to be negligible in the first ex-vivo trials^13^. On the other hand, as far as the patient is concerned, OLINDA calculations suggest that an effective dose of about 25 mSv is expected in the considered scenario if the patient is catheterized after the injection, which is easily the case given the surgical intervention.

## Conclusion

In this work we studied the applicability of an innovative *β*^−^-RGS technique to pNETs with ^90^Y-DOTATOC, evaluating its uptake with a retrospective study on ^68^Ga-DOTATOC PET images of 30 patients at Fondazione Policlinico Universitario A. Gemelli IRCCS in Rome. The technique has been demonstrated to be feasible by injecting the patient with 1.5 MBq/kg of ^90^Y-DOTATOC 24 h before surgery, with a sampling time for each spot of the order of few seconds.

Indeed, despite the expected variability among patients, only in two cases the technique to be effective would have required either a mildly increase in the amount of administered activity or a longer sampling time. For all the other cases, we found that the patients’ SUV and TNR are compatible with the application of the proposed technique.

In any case, in the application protocol we propose, we foresee a preliminary PET scan with ^68^Ga-DOTATOC to verify in advance that the technique could be effectively applied to the patient. This would allow also to tailor the injected activity accordingly to the individual uptake as discussed in a previous publication^14^.

To verify the expected performances of the technique, ex-vivo tests on excised pNETs specimens are needed and foreseen for the next future. At the same time, we are working on improving the detector to lower significantly the minimum detectable energy of the electrons^27,28^. Such an improvement would in fact lower either the probing time, or the administered activity.

## Data Availability

The data that support the findings of this study are available from the corresponding author on reasonable request.
